# 2896. Oral PrEP Uptake among HVTN 704/HPTN 085 Participants: Implications for the Future of Biomedical HIV Prevention Trials

**DOI:** 10.1093/ofid/ofad500.167

**Published:** 2023-11-27

**Authors:** Valeria D Cantos, Moni Neradilek, Kevin Gillespie, Yunda Huang, Allan C deCamp, Shelly T Karuna, Srilatha Edupuganti, Carlos del Rio, Valdilea Veloso, Myron S Cohen, Deborah J Donnell, Lawrence Corey, Alison C Roxby, Colleen F Kelley

**Affiliations:** Emory University, Atlanta, GA; SCHARP (Fred Hutch Cancer Center), Seattle, Washington; Fred Hutchinson Cancer Research Center, Seattle, Washington; Fred Hutch, Seattle, Washington; Fred Hutchinson Cancer Research Center, Seattle, Washington; Greenlight Bioscience, Seattle, Washington; Emory University School of Medicine, Decatur, GA; Emory University School of Medicine, Division of Infectious Diseases, Decatur, Georgia; Instituto Nacional de Infectologia Evandro Chagas-Fiocruz, Rio de Janeiro, Rio de Janeiro, Brazil; University of North Carolina, Chapel Hill, North Carolina; Fred Hutchinson Cancer Research Center, Seattle, Washington; Fred Hutchinson Cancer Research Center, Seattle, Washington; University of Washington, Seattle, Washington; Emory University, Atlanta, GA

## Abstract

**Background:**

The HVTN 704/HPTN 085 clinical trial (AMP study) assessed the efficacy of VRC01 broadly neutralizing antibody infusions for HIV prevention. It offered access to oral pre-exposure prophylaxis (PrEP) at no-cost to all participants as standard of HIV prevention care. Understanding PrEP initiation among trial participants and describing its impact on study endpoints can guide future HIV prevention trial design.

**Methods:**

We characterized sociodemographic and geographic features of PrEP initiation (by self-report) using descriptive statistics. We used Cox models to identify factors associated with PrEP initiation and the association between PrEP initiation (as a time-dependent exposure) and HIV incidence.

**Results:**

Of 2221 participants off PrEP at enrollment, 31.8% initiated PrEP during the trial. Median time to PrEP initiation was 60 days (range 1-673 days). Brazil had the highest proportion of PrEP initiation among their enrolled participants (83.2%) and Peru had the lowest (5.3%). In US sites, 54.2% initiated PrEP. Southern US sites had the highest proportion of PrEP initiation (68.8%) and the Midwest had the lowest (28.4%).

In multivariate analysis, prior PrEP use [HR (95% CI) = 2.2 (1.3-4.0)] was associated with PrEP initiation. Participants from Switzerland [0.5 (0.3-1.0)] and Peru [0.08 (0.06-0.1)] had lower likelihood of PrEP initiation compared to the US, while participants from Brazil had higher likelihood [2.6 (2.0-3.3)]. Age, race, gender, and sexual orientation were not associated with PrEP initiation. HIV incidence rate was 3.6/100 person-years (PY) before PrEP initiation, compared to 1.2/100 PY after PrEP initiation. PrEP initiators had 58% less risk of acquiring HIV after adjusting for behavioral risk score and treatment arm. In the US, higher local PrEP-to-need ratio was also associated with lower PrEP initiation [0.9 per 5 units (0.8-1.0)].

**Conclusion:**

Providing PrEP access to AMP participants reduced HIV acquisition among PrEP initiators. Study endpoints were still met due to heterogenous regional PrEP uptake and lower PrEP effectiveness than expected. These data suggest that offering oral PrEP to HIV prevention trial participants as standard of care is a feasible option to consider for future efficacy HIV prevention trial design.

**Disclosures:**

**Valeria D. Cantos, MD**, Gilead Sciences: Grant/Research Support **Srilatha Edupuganti, MD MPH FIDSA**, Sanofi: Grant/Research Support **Colleen F. Kelley, MD, MPH**, Gilead Sciences: Grant/Research Support|Humanigen: Grant/Research Support|Moderna, Inc.: Grant/Research Support|Novavax, Inc: Grant/Research Support|Viiv Healthcare: Grant/Research Support

